# Identifying low test-taking effort during low-stakes tests with the new Test-taking Effort Short Scale (TESS) – development and psychometrics

**DOI:** 10.1186/s12909-018-1196-0

**Published:** 2018-05-08

**Authors:** Katrin Schüttpelz-Brauns, Martina Kadmon, Claudia Kiessling, Yassin Karay, Margarita Gestmann, Juliane E. Kämmer

**Affiliations:** 1 Medical Faculty Mannheim at Heidelberg University, Theodor-Kutzer-Ufer 1-3, 68167 Mannheim, Germany; 2 Carl von Ossietzky University Oldenburg, Carl-von-Ossietzky-Straße 9-11, 26129 Oldenburg, Germany; 3 Brandenburg Medical School Theodor Fontane, Fehrbelliner Straße 38, 16816 Neuruppin, Germany; 4 Medical Faculty, University of Cologne, Joseph-Stelzmann-Straße 20 (Building 42), 50931 Cologne, Germany; 5 Medical Faculty, University of Duisburg-Essen, Hufelandstraße 55, 45147 Essen, Germany; 6 AG Progress Test Medizin, Charité Universitätsmedizin Berlin, Hannoversche Straße 19, 10115 Berlin, Germany; 7 Center for Adaptive Rationality, Max Planck Institute for Human Development, Lentzeallee 94, 14195 Berlin, Germany

**Keywords:** Nonconsequential progress testing, Psychometrics, Self-assessment, Short scale, Test-taking effort, Validation study

## Abstract

**Background:**

Low-stakes tests are becoming increasingly important in international assessments of educational progress, and the validity of these results is essential especially as these results are often used for benchmarking. Test scores in these tests not only mirror students’ ability but also depend on their test-taking effort. One way to obtain more valid scores from participating samples is to identify test-takers with low test-taking effort and to exclude them from further analyses. Self-assessment is a convenient and quick way of measuring test-taking effort. We present the newly developed Test-taking Effort Short Scale (TESS), which comprises three items measuring attainment value/intrinsic value, utility value, and perceived benefits, respectively.

**Methods:**

In a multicenter validation study with *N* = 1837 medical students sitting a low-stakes progress test we analyzed item and test statistics including construct and external validity.

**Results:**

TESS showed very good psychometric properties. We propose an approach using stanine norms to determine a cutoff value for identifying participants with low test-taking effort.

**Conclusion:**

With just three items, TESS is shorter than most established self-assessment scales; it is thus suited for administration after low-stakes progress testing. However, further studies are necessary to establish its suitability for routine usage in assessment outside progress testing.

**Electronic supplementary material:**

The online version of this article (10.1186/s12909-018-1196-0) contains supplementary material, which is available to authorized users.

## Background

### Test-taking effort

Large-scale assessments like the Progress in Reading Literacy Study (PIRLS), the Trends in International Mathematics and Science Study (TIMSS) (see http://timss.bc.edu), the US National Assessment of Educational Progress (NAEP) [1], and the Program for International Student Assessment (PISA) [2, 3] are used as benchmarks of educational systems and student achievement worldwide. This benchmarking process has a substantial impact on the reputation of educational systems, as well as on educational reform, policy-making, and resource allocation [4, 5]. In Germany, for example, the results of PISA 2000 sparked a broad public debate about the German school system and led to the implementation of major reforms [4] despite these large-scale assessments being low-stakes for the participants. Participants did not face any negative consequences if they didn’t perform at their best. Yet, students’ achievement in any test not only mirrors their underlying ability but also depends on their test-taking effort [6, 7], the “extent to which an examinee gives his or her best effort” [8]. Test scores, therefore, do not only reflect ability but also test-taking effort, with corresponding effects on test validity [9, 10].

In high-stakes testing, the consequences for test-takers can be significant, potentially leading to high test-taking effort and, in turn, better performance. In low-stakes testing, in contrast, the test result has no consequences for test-takers [9], which may decrease the subjective task value, resulting in lower motivation or test-taking effort and, in turn, lower performance [10, 11]. Nevertheless, low-stakes testing is becoming increasingly important, not only

- (1) in large-scale international assessments (e.g., NAEP, TIMSS, PISA) [10, 12], but also
- (2) in the evaluation of curricula [9],
- (3) in piloting new test items for high-stakes testing, and
- (4) in empirical research [9].

Progress testing in medical education is an example of low-stakes testing. In Germany and Austria, for example, progress tests are used as a means of formative assessment [13] and are therefore low stakes. Progress tests are administered repeatedly during undergraduate training (e.g., once per semester), with students of all semesters undergoing the same test. In Germany and Austria, for example, 15 medical faculties administer the Berlin Progress Test (BPT) [13, 14] with about 10,000 students twice a year. Faculties use the information gathered to evaluate, develop, and compare their curricula and to provide students with feedback on their current level of knowledge and development [13–17]. As the conclusions drawn from these tests may be far reaching, it is important for faculties and researchers to keep track of test-taking effort and to potentially exclude participants with low test-taking effort from their analyses.

### How to reliably measure test-taking effort

Currently, there are three approaches to measuring test-taking effort. The first is to measure *response time to test items*, under the assumption that participants with low test-taking effort will take less time to think about their answers and will therefore answer questions faster than participants with high test-taking effort [9]. Measuring response time is convenient in computer-based assessment. However, it does not differentiate between low test-taking effort and test-takers with high expertise, who are able to identify keywords in the question and decide within seconds whether they can answer it or not. A second approach is *appropriateness measurement*, whereby the probability of answer patterns is calculated on the basis of either estimated empirical models or theoretical parametric/nonparametric item response models [18–20]. Lack of fit between a test-taker’s answer pattern and the model is then attributed to lack of motivation and low test-taking effort [9]. There are, however, two weaknesses to this approach: (1) A misfit between test-taker data and model does not necessarily imply a lack of motivation and test-taking effort but may also imply differential item functioning, i.e. test-takers with different learning strategies or experiences may show distinct response patterns despite exerting the same high test-taking effort. In this case, some test-takers would be erroneously identified as showing low effort. (2) Calculating misfit of a test-takers’ answer pattern in large samples requires sophisticated statistical skills. Whereas this expertise is in place in large-scale assessments, it is not always present in experimental/social research.

In the third and currently most widely used approach, test-taking effort is measured with *self-assessment scales*. In contrast to the response time approach, self-assessment allows fast experts to be differentiated from test-takers with low test-taking effort. Furthermore, it does not require sophisticated statistical skills. The drawback of this third approach is that, like any other self-report method, it is vulnerable to motivational processes. Long self-reports, for example, may decrease the motivation for meaningful answers or to answer at all. Thus, not responding can in itself be an expression of low test-taking effort [21]. Self-assessment scales therefore need to be very short to mitigate motivational effects on answers. An overview of published self-assessment scales is given in Table 1. However, the established instruments are rather long, with an average of 15 items. As large-scale assessments often take several hours, any test effort self-reports administered immediately afterwards should be short, in order to prevent low compliance or low motivation resulting in invalid results [9]. The shortest instrument, the Effort Thermometer (Table 1), has just three items but is not suited for filtering out test-takers with low test-taking effort as its purpose is to measure intraindividual differences of effort in assessments with different stakes; additionally, it has no theoretical framework and no reported psychometrics.

**Table 1:** Self-report measures of test-taking effort

Instrument	No. items	Subscales	What is measured	Psychometrics
Effort Thermometer [12]	3 10-point Likert items	No subscales	Individual test effort, anchored against a personal situation in which maximum effort was applied	Not reported
Online Motivation Questionnaire (OMQ) [47]	32 4-point Likert items	Mood scale Self-efficacy Success expectancy Perceived utility Task attraction Intended efforts Task anxiety scale	Test-taking effort in the context of performance assessment (part 1: pre-test, part 2: post-test)	Confirmatory factor analysis revealed that seven factors could be distinguished empirically; proven validity and acceptable reliability
Questionnaire of Current Motivation (QCM) [48]	18 7-point Likert items	Situational interest Anxiety Challenge Probability of success	Current motivation during a learning situation	Sufficient to excellent reliability2 Proven validity
Student Opinion Scale (SOS)	10 5-point Likert items	Importance Effort	Motivation, administered as a post-test after students have completed achievement tests [49]	Proven validity and good to excellent reliability [50]

### Construction of the test-taking effort short scale (TESS)

We used a theory-driven approach to test construction to develop TESS [22].

Expectancy-value theory as conceptual framework to explain test-taking effort.

Expectancy-value theory—a well-established and empirically validated psychological theory—has already been used to construct self-report instruments measuring test-taking effort, such as the Student Opinion Scale (see Table 1). According to expectancy-value theory, achievement-related choices (e.g., effort, performance, or persistence) depend on the test-taker’s expectation of success and the subjective task value [23]. Subjective task value consists of four components:

1. *Attainment value* is the importance of doing well.
2. *Intrinsic value* is the enjoyment of engaging with the task.
3. *Utility value* depends on how well a task fits into an individual’s future plans. In low-stakes assessment, it may depend on how relevant a test is to a test-taker—in terms of being useful for assessing one’s learning progress, for instance.
4. The variable *cost* assesses to what extent engaging in one task limits access to other activities, as well as emotional costs.

Studies with students from elementary and secondary schools, colleges, and universities have provided empirical evidence for expectancy–value theory. For example, Trautwein et al. showed that the expectancy and value components predicted achievement in secondary school students [24] and Chiu and Wang found that they predicted continued use of web-based learning even when desisting had no consequences [25]. Perceived utility value has been shown to predict performance (e.g., course points in an English class, [26]; or correctly solved multiplication problems [27]). Likewise, the variables usefulness and importance have significantly predicted test-taking effort and performance in several tests with undergraduate students [28].

#### Constructing content-valid items

In low-stakes assessment, wanting to achieve the best possible results is an expression of attainment value as well as intrinsic value. Item 1 of TESS (see Table 2) measures this factor. Utility value is captured by item 2, which asks how useful the test is to the student (see Table 2). If low-stakes tests fit into an educational program, their perceived costs will be lower, as they will be seen as equally important as other parts of the program. Item 3 taps this factor by asking students whether the test is a valuable part of their education (see Table 2).

**Table 2:** Item statistics

Item	*M*	*SD*	*p*_*i*_	*r*_*cis*_	*H*_*is*_
1. I want to achieve the best possible results in the test. [German: Ich möchte beim PTM die bestmöglichen Ergebnisse erreichen.]	3.14	1.36	0.47	0.70	0.68
2. I think the progress test is useful. [German: Ich finde den PTM sinnvoll.]	3.27	1.35	0.50	0.76	0.72
3. The test is a valuable part of my education. [German: Der PTM ist ein wertvoller Teil meines Studiums.]	2.48	1.25	0.31	0.75	0.73

*M* mean, *SD* standard deviation, *p*_*i*_ difficulty, *r*_*cis*_ discriminatory power (part–whole corrected), *H*_*is*_ Mokken homogeneity coefficient of item with scale

All three items were constructed as 5-point Likert items with the anchors 1 “fully disagree” to 5 “fully agree.” Thus, TESS consists of three items, the first measuring attainment value and intrinsic value, the second measuring utility value, and the third measuring perceived benefits (i.e., reverse-coded costs).

### Aims

Our first aim was to develop a short test-effort self-assessment scale that is capable of measuring test effort in low-stakes testing with high reliability and validity. Our second aim was to conduct a validation study for the scale developed.

## Methods

To validate the newly developed TESS, we conducted a study with *N* = 1837 medical students involved in regular progress testing at eight medical schools in Germany and Austria. We analyzed item and test statistics of TESS, i.e. mean, standard deviation, difficulty, and discrimination as well as reliability, homogeneity, construct validity, and external validity. We standardized values to identify participants with low test-taking effort. Additionally, we analyzed response rates to determine whether non-response to TESS is diagnostic of low test-taking effort (see also [21].

### Sample

All students who participated in the Berlin Progress Test (BPT) [13, 14] at eight medical schools in Germany and Austria in winter semester 2015/2016 (*N* = 4624) were invited to participate in this study. Of these, 1837 students (40%) completed the questionnaire and were included in the validation study. Of the 1654 participants who reported their sex, 639 were male (39%) and 1015 were female (61%); 183 participants did not report their sex. Mean age was 23.81 years (SD = 3.99, range: 17–56). The demographic distribution of our sample resembled that of the population of medical students in Germany (mean age M = 23.7, sex distribution: 61% female [29]. We therefore consider our sample to be representative of the medical student population.

### Procedure

Students were invited to voluntarily complete TESS as part of a more extensive study not pertinent to this manuscript administered after the BPT. The 9-page questionnaire was administered in the same format as the progress test, namely in either computer-based or paper-based format. In total, 857 computer-based (47%) and 980 paper-based (53%) questionnaires were completed. The Ethical Review Board of Medical Faculty Mannheim, Heidelberg University, approved the study (2015-542 N-MA).

### Material

TESS was used to measure students’ test-taking effort in the BPT via self-assessment. TESS is included in the supplementary material (see Additional file 1). Further measures were included in the post-test study to assess the construct validity and external validity of TESS.

#### Construct validity

Following Campbell and Fiske [30], we assessed the construct validity of TESS by determining its convergent and discriminant validity. Convergent validity assesses the degree to which different tests designed to measure the same construct are, in fact, related. Discriminant validity assesses the degree to which tests designed to measure different constructs are, in fact, unrelated.

To determine *convergent validity*, we used the following established and new measures to assess test-takers’ intrinsic motivation (relates to item 1), the perceived usefulness of the BPT (relates to item 2) and its perceived benefits (relates to item 3). High correlations with the respective TESS item would indicate convergent validity on the item level.

*Intrinsic motivation* was measured using the Short Scale of Intrinsic Motivation, which consists of twelve 5-point Likert items and has been shown to be reliable and valid [31]. A sample item is “I found the BPT very interesting” [German: “Ich fand den PTM sehr interessant”].

*Perceived usefulness* was measured with a German translation of the Perceived Usefulness Scale, which consists of seven 5-point Likert items and has shown excellent psychometric properties in two studies [32]. A sample item is “I expect the BPT to be useful for learning” [German: “Ich erwarte, dass mir der PTM beim Lernen nützlich sein wird”].

*Perceived benefits* were assessed with a self-constructed 5-point Likert item targeting costs: “To what extent do you feel that sitting the BPT keeps you from your other duties?” [German: “In welchem Maße werden Sie durch den PTM in Ihren anderen Verpflichtungen eingeschränkt?”].

To determine *discriminant validity*, we additionally assessed a variable that is conceptually independent of test-taking effort but still related to test performance, namely, learning strategy use. Learning strategies are action plans used to control and monitor one’s learning. They are used to reach learning goals efficiently and are oriented towards learning and not towards taking a test. As test-taking effort depends on the situation [33], it should not strongly relate to the learning strategy use.

*Learning strategy use* was measured with the Repetition scale of the Learning Strategies in Undergraduate Training test (German: Lernstrategien im Studium, LIST), which consists of eight 6-point Likert items and has been shown to be reliable and valid [34]. A sample item is “I learn the content of texts by reading them again and again” [German: “Ich präge mir den Lernstoff von Texten durch Wiederholen ein”].

#### External validity

To obtain external criteria of participants’ test-taking effort, we asked them to report the *test score* and *test time* of their previous BPT. Both measures would be expected to be low if test-taking effort was low [9, 14]. The BPT test score is calculated as the number of correct answers minus the number of incorrect answers. Test time is the time taken on the test.

All data used in this study can be found in the supplementary material (see Additional file 2).

### Response rates

As completion of TESS was voluntary, participants could in principle answer between 0 and 3 TESS items. Response rates indeed varied between 0 and 3 TESS items. For further analyses, responders were defined as those with 3 completed TESS items; non-responders as those with 0 completed items (despite participating in the study). We excluded all participants who answered 1 or 2 items from our analysis of response rates.

In order to analyze whether non-response to TESS was diagnostic of low test-taking effort, we compared the BPT test times and test scores of non-responders with those of responders with low TESS scores (objectively indicating low test-taking effort) and with those of responders with high TESS scores. If findings showed that non-responders do not differ from participants with low TESS scores in terms of the BPT variables, but that they do differ from participants with high TESS scores, this will indicate that non-response to TESS is diagnostic of low test-taking effort.

### Statistical analysis

For each of the three TESS items, we determined the mean, standard deviation, difficulty, discriminatory power, and item–scale homogeneity. Difficulty *p*_*i*_ per item was calculated as the sum of squared scores divided by the number of participants multiplied by the squared maximum score (in the case of a 5-point Likert item = 25) [35].

Corrected item–scale correlation (*r*_cis_) was used to compute item discrimination. This correlation is categorized as moderate if .30 ≤ *r*_cis_ < .50 and as high if r_cis_ ≥ .50 [36]. Cronbach’s alpha (α) was used to estimate the reliability of TESS. Reliability is categorized as low if α < .80, moderate if .80 ≤ α < .90, and high if α ≥ .90 [36]. Scale homogeneity (*H*_s_) as well as item–scale homogeneity (H_is_) was analyzed using Mokken scale analysis, which analyzes the goodness of fit to the Guttman structure. A scale is unscalable if *H*_s_ < .30, weak if .30 ≤ *H*_s_ < .40, medium if .40 ≤ *H*_s_ < .50, and strong if *H* ≥ .50. The same applies to *H*_is_ [37].

To determine convergent validity, we calculated Spearman rank correlations between the three TESS items and the Short Scale of Intrinsic Motivation, the Perceived Usefulness Scale, and the self-constructed item tapping the costs of the BPT, respectively. To estimate discriminant validity, we calculated product-moment correlations between the TESS score and the score on the Repetition scale of the LIST. Effect size *r*^2^ was calculated and effects were categorized as large for *r*^2^ ≥ 0.25, as moderate for 0.09 ≤ *r*^2^ < 0.25, and as small for 0.01 ≤ *r*^2^ < 0.09 [38].

External validity was estimated by group comparisons of low/high BPT test scores and short/long BPT test time. To this end, participants were ranked with respect to each of those variables. Students in the top and bottom 20% of the sample were then compared with respect to their indicated test effort with one-way analysis of variance.

In low-stakes assessment, a binominal distribution of test-taking effort scores can be expected, with a first peak at very low scores for participants who did not take the test seriously and a second peak at average scores. Therefore, we used stanine norms—which are directly derived from percentile rank—to determine a cutoff value for participants with low test-taking effort. Stanine values of 1 and 2 mean arbitrary values.

Additionally, we used one-way ANOVAs with post hoc tests (Scheffé tests) to compare the non-responders with the groups of responders with low vs. high test-taking effort according to stanine standardization. Effects were categorized as large for η^2^ ≥ 0.1379, as medium for 0.0588 ≤ η^2^ < 0.1379 and, and as small for 0.0099 ≤ η^2^ < 0.0588 [38].

IBM Statistics SPSS 23 was used to calculate the results. The R package Mokken [39] was used to compute the Mokken homogeneity coefficient.

## Results

### Item statistics

Item means ranged from 2.48 to 3.27 with an average of 2.96 and a standard deviation of 1.32 (see Table 2). Item difficulty ranged between medium (item 3) and high (item 2). The discriminatory power and item–scale homogeneity of each item was high.

### Test statistics

#### Reliability and homogeneity

The reliability of TESS was moderate with Cronbach’s α of 0.86. The Mokken homogeneity of the TESS scale was high (*H* = 0.71).

#### Construct validity

The correlations of the three TESS items with the corresponding external criteria of convergent validity were moderate (for item 3) to large (for items 1 and 2). The TESS score correlated significantly but with no practical effect with the Repetition scale of the LIST, providing evidence for discriminant validity (see Table 3).

**Table 3:** Correlations of single TESS items and the TESS score with external criteria

Internal criterion	External criterion	*N*	*r*	*p*	*r* ^2^
Convergent validity					
TESS item 1	Short Scale of Intrinsic Motivation [31]	1333	.52	<.001	.27
TESS item 2	Perceived Usefulness Scale [32] (German translation)	1377	.57	< .001	.32
TESS item 3	Cost^#^	1380	−.34	< .001	.12
Discriminant validity					
TESS score	Repetition scale of the LIST [34]	1195	.06	<.05	.00

*N* number of participants, *r* product-moment correlation, *p p*-value, *r*^2^ effect size, ^#^ item “To what extent do you feel that sitting the BPT keeps you from your other duties?” (reverse coded)

#### External validity

Ranked in terms of BPT test time, the lower 20% of participants (*N* = 314; fast performers) needed 41 min (SD = 21 min) on average to complete the progress test; the upper 20% of participants (*N* = 262; slow performers) needed 2 h and 40 min (SD = 17 min) of the maximum of 3 h an examinee can expend on the BPT. These two groups differed significantly in terms of their TESS scores, with fast performers having lower TESS scores than slow performers (see Table 4).

**Table 4:** TESS scores in extreme groups

		TESS values		
External criterion	Group	*M* (*SD*)	*N*	ANOVA
BPT test time	Fast performers^a^	2.29 (1.06)	257	*F*(1) = 258.70; *p* < 0.001; η^2^ = 0.35
	Slow performers^b^	3.70 (0.87)	237	
BPT test score	Poor performers^c^	2.66 (1.25)	100	*F*(1) = 27.03; *p* < 0.001; η^2^ = 0.12
	High performers^d^	3.50 (1.08)	110	

*N* number of participants (the difference to the *N*s reported in the text is due to the fact that not all participants belonging to the extreme groups also reported their test time and test score), *M* mean TESS scores; *SD*: standard deviation, *BPT* Berlin Progress Test

^a^M = 0:41, SD = 0:21 to take the test

^b^M = 2:40, SD = 0:17 to take the test

^c^test score of M = 1.46, SD = 2.87

^d^test score of M = 92.46, SD = 30.11

Ranked in terms of BPT test scores, the lower 20% of participants (*N* = 126; poor performers) achieved an average score of 1.46 (SD = 2.87); the upper 20% (*N* = 130; high performers) an average score of 92.46 (SD = 30.11). Poor performers had significantly lower TESS scores than high performers (see Table 4).

#### Standardization

TESS scores with a stanine norm of 1 or 2, indicating percentile ranks of 0 to 11%, can be interpreted as signaling low test-taking effort. TESS scores with a stanine norm of 8 or 9, indicating percentile ranks of 90 to 100%, can be interpreted as indicating high test-taking effort [40]. As shown in Table 5, a TESS score of 1 corresponded to a stanine score of 1 or 2, indicating low test-taking effort, whereas TESS scores higher than 4 corresponded to a stanine score of 8 or 9, indicating high test-taking effort. A TESS score of 1 refers to an individual who chose 1 on the 5-point Likert-scale for all three of the TESS items.

**Table 5:** Percentile ranks of TESS scores and corresponding stanine values

TESS score	*N*	Percentile rank	Stanine	Interpretation
1	157	11.4	1, 2	Low TTE
1.33	55	15.4	3	
1.67	67	20.3	3	
2	95	27.2	4	
2.33	88	33.6	4	
2.67	119	42.3	5	
3	141	52.6	5	
3.33	157	64	6	
3.67	136	73.9	6	
4	144	84.4	7	
4.33	81	90.3	8	High TTE
4.67	60	94.7	8	High TTE
5	73	100	9	High TTE

*TTE* test-taking effort

#### Response rates

**Fig. 1:**
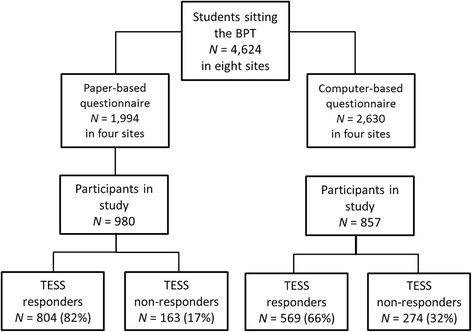
Flowchart showing participant numbers separately for computer- and paper-based administration; TESS responders are defined as participants who answered all three TESS items; non-responders are defined as participants who did not answer any TESS items (despite participating in the study)

Of the 1837 study participants, 1373 answered all three TESS questions (75%; i.e., responders), whereas 437 did not answer any (24%; i.e., non-responders). Twenty-seven participants answered one or two TESS questions. With respect to the administration format, 804 (82%) of the 980 participants who did the test on paper answered all three TESS questions and 163 (17%) did not answer any, whereas only 569 (66%) of the 857 participants who did the test on a computer answered all three questions and 274 (32%) did not answer any (see Fig. 1). Twenty-seven participants answered one or two TESS questions, 13 of them on paper and 14 on computer.

Mean BPT test time in non-responders was significantly higher than in participants with low test-taking effort and significantly lower than in participants with high test-taking effort (see Table 6). In terms of mean BPT test scores, in contrast, there was no significant difference between the non-responders and the participants with either low or high test-taking effort (see Table 6).

**Table 6:** Comparison of different groups of (non)-responding in TESS

External criterion	Group	*N*	*M* (*SD*)	ANOVA	Scheffé test
(A) BPT test time (hours:minutes)	Non-responders (G1)	122	1:22 (0:53)	F(2) = 85.40; *p* < 0.01, η^2^ = 0.29	G2 < G1 < G3
	Low TTE (G2)	100	0:58 (0:40)		
	High TTE (G3)	198	2:01 (0:34)		
(B) BPT test score	Non-responders (G1)	71	35.77 (45.14)	F(2) = 9.31, p < 0.01, η^2^ = 0.08	G1 = G2 and G1 = G3
	Low TTE (G2)	52	22.04 (36.08)		
	High TTE (G3)	106	49.27 (33.44)		

Comparison of non-responders in TESS (G1) with responders with low test-taking effort (G2) and responders with high test-taking effort (G3), as defined by (A) BPT test scores or (B) BPT test time

*N* number of participants, *M* mean, *SD* Standard deviation, *TTE* test-taking effort, low vs. high TTE determined by stanine standardization, see Table 5

## Discussion

Performance in low-stakes tests occasionally depends on examinees’ test-taking effort. One approach to obtaining valid scores is to identify examinees with low test-taking effort and to exclude their answers from analyses. In this article, we introduced the Test-taking Effort Short Scale (TESS), a short self-assessment scale designed to measure test-taking effort in low-stakes progress testing, in particular. We assessed the scale’s psychometric properties in a multicenter validation study (eight medical schools) with *N* = 1837 medical students taking a regular progress test.

TESS was developed on the basis of expectancy–value theory [23] and adapted to the special situation of low-stakes assessment. With just three 5-point Likert items, it is much shorter than most other instruments measuring test-taking effort. This brevity is of considerable advantage in lengthy low-stakes assessments, helping to combat decreasing motivation and thus to increase response rates.

Our findings showed that TESS has very good psychometric properties. Reliability was moderate and scale homogeneity proved to be high. Convergent validity was moderate to high. The reason for item 3 showing only moderate convergent validity may be that, lacking a standardized scale to measure the cost of test-taking, we used a one-item measure to determine the convergent validity of the TESS costs item. This one-item measure directly assesses the cost of taking a low-stakes test, but its validity and reliability are unknown. The TESS items were not significantly related to any of the discriminant variables. Due to large sample sizes TESS score correlated significantly with the external correlation. However correlations were so small that there is no indication for a practical effect. Our findings thus confirmed the construct validity of TESS. Furthermore, our stanine standardization approach to identifying participants with low test-taking effort provided evidence for the external validity of TESS. Our sample was representative of the population of medical students in Germany with respect to age and sex; standardizing was thus justified.

Seventy-five percent of the participants in this study answered all three TESS items. Our response rate analysis showed that non-responders could not be allocated to either the high or the low test-taking effort group in terms of their test times and test scores. Thus, our sample included a rather high number of students whose test-taking effort could not be determined. A reason for this may be that TESS was embedded in an extensive questionnaire administered after a test lasting up to 3 h (BPT). Had TESS been administered alone, the response rate might have been higher. Indeed, 91% of examinees typically answer the voluntary evaluation form regularly administered after the BPT, which comprises 4 multiple choice items. Thus, further investigations are needed before our results can be generalized to routine usage in assessment within and beyond medical progress testing.

With respect to administration format, we found that there were fewer non-responders in the paper-based format than in the computer-based format. Several studies comparing computer-based vs. paper-based evaluation of teaching have yielded similar results [41, 42]. One reason for this difference could be survey fatigue in the context of online surveys [43]. As computer-based assessment becomes increasingly widespread, further studies are needed to identify factors influencing response rates in computer-based assessment.

Certain limitations of our study warrant consideration. First, like every self-assessment instrument, TESS may be subject to social desirability bias. A 9-page questionnaire added to the assessment may have impacted TESS-related data beyond survey fatigue. Nevertheless, TESS proved to have excellent psychometric properties, is able to differentiate between low test-taking effort and high expertise, and does not require a high level of statistical skill. Second, data on the external criteria (BPT test time and BPT test scores) were collected as self-reports on past test participation. If less motivated students respond carelessly, such self-report measures may lack accuracy [8]. Objective measurement of the actual test-taking time and score may have provided more valid external criteria. Our approach may also be less sensitive in terms of identifying low test-taking effort via TESS. Due to privacy protection in this study, however, objective measures of BPT test time and BPT test score were not available. A second study is planned to circumvent these drawbacks.

The three-item TESS is suitable for administration after low-stakes progress tests. A TESS score of 1 identifies participants with low test-taking effort, whose results therefore threaten the validity of the assessment. Using TESS rather than response time or appropriateness measurement to statistically identify test-takers with low test-taking effort shows test-takers that administrators are concerned about the problem of low test-taking effort. In our experience, test-takers with average test-taking effort are likely to increase their effort if they know that the results of a low-stakes assessment will not be negatively influenced by test-takers with low test-taking effort.

Further studies are needed to investigate the following aspects:

- applicability of TESS in low-stakes assessment other than progress testing and after translation into other languages;
- standardization of scores in other low-stakes assessments, contexts, and populations;
- response rates when TESS is the only instrument administered after a low-stakes assessment;
- reasons for lower response rates in computer-based than paper-based assessment;
- sensitivity and specificity of low test-taking effort as measured by TESS with an objective measurement of low test-taking effort.

## Conclusion

The results of large-scale assessments can have a considerable impact on education policy and practice [5]. As large-scale assessment is usually low stakes, individual test-takers’ performance may be influenced not only by their ability but by their test-taking effort [44]. Using a reliable and parsimonious tool such as TESS to filter out participants with low test-taking effort could be a good way of improving the validity of the conclusions drawn from large-scale assessments. Furthermore, as health professions education moves towards more formative assessment strategies (van der Vleuten, programmatic assessment [45, 46]) measures that facilitate assessing the rigor of test questions used in formative assessments will be needed in many settings.

## Additional files

Additional file 1: Questionnaire of TESS. (DOCX 18 kb)

Additional file 2: Raw data with all variables used in this study. (XLSX 150 kb)

## Abbreviations

BPT: Berlin progress test
TESS: Test-taking effort short scale
TTE: Test-taking effort
