# Supplementary material for: Identifying low test-taking effort during low-stakes tests with the new Test-taking Effort Short Scale (TESS) – development and psychometrics
Source: BMC Med Educ. 2018 May 8;18:101. doi: 10.1186/s12909-018-1196-0 (PMC5941641; doi:10.1186/s12909-018-1196-0)
Supplement: Supplementary file 1 — Questionnaire of TESS. (DOCX 18 kb) [file 12909_2018_1196_MOESM1_ESM.docx]

Test-effort Short Scale (TESS)

|  | Fully disagree |  |  |  | Fully agree |
| --- | --- | --- | --- | --- | --- |
| I want to achieve the best possible results in the test. [German: Ich möchte beim PTM die bestmöglichen Ergebnisse erreichen.] | 🞏 | 🞏 | 🞏 | 🞏 | 🞏 |
| I think the progress test is useful. [German: Ich finde den PTM sinnvoll.] | 🞏 | 🞏 | 🞏 | 🞏 | 🞏 |
| The test is a valuable part of my education. [German: Der PTM ist ein wertvoller Teil meines Studiums.] | 🞏 | 🞏 | 🞏 | 🞏 | 🞏 |
